# Risk of Infective Endocarditis Following Invasive Dental Procedures: A Systematic Review and Meta-Analysis

**DOI:** 10.3389/phrs.2024.1607684

**Published:** 2025-01-08

**Authors:** Zhumakyz Kussainova, Mukhit Kulmaganbetov, Askar Abiltayev, Tolkyn Bulegenov, Islam Salikhanov

**Affiliations:** ^1^ Department of Public Health, Semey State Medical University, Semey, Kazakhstan; ^2^ Centre for Eye and Vision Research Limited (CEVR), Hong Kong, Hong Kong SAR, China; ^3^ Department of Surgery, University Hospital of Basel, Basel, Switzerland

**Keywords:** infective endocarditis, invasive dental procedures, tooth extraction, endodontic treatment, oral surgery

## Abstract

**Objectives:**

This systematic review and meta-analysis aimed to synthesize evidence and determine the association between IE and dental procedures, including invasive and non-invasive procedures.

**Methods:**

We conducted a systematic search of PubMed, Google Scholar, and Cochrane databases for studies involving procedures such as tooth extraction, scaling, endodontic treatment, oral surgery, and periodontal treatment involving individuals aged ≥15 years. The primary outcome was the incidence of IE following these procedures.

**Results:**

An association was found between IE and invasive dental procedures (OR 1.49, 95% CI 1.25–1.76; p < 0.00001). Subgroup analysis showed an increased risk of IE following tooth extraction (OR 2.73, 95% CI 1.46–5.11; p = 0.002) and oral surgery (OR 6.33, 95% CI 2.43–16.49; p = 0.0002) in high-risk patients.

**Conclusion:**

Our study found a strong association between invasive dental procedures and increased IE risk, particularly for tooth extraction and oral surgery in high-risk individuals.

**Systematic Review Registration:**

https://www.crd.york.ac.uk/prospero/display_record.php?ID=CRD42023488546, Identifier CRD42023488546.

## Introduction

Infective endocarditis (IE) is a rare disease with an incidence rate of 7.0–14.3 cases per 100,000 and a 3-month mortality rate nearing 40% among diagnosed patients [1]. The high mortality is largely attributed to the necessity for emergent surgical interventions in numerous cases, driven by the emergence of severe complications and the presence of infections caused by resistant and virulent pathogens [2]. The risks associated with IE are linked to bacteremia, which can arise not only from invasive dental procedures but also from routine daily activities such as flossing, brushing teeth, and chewing [3]. Despite these associations, establishing a direct causal link between IE and invasive dental procedures remains challenging, as definitive conclusions cannot be drawn from individual observational studies alone.

A recent systematic review and meta-analysis established an association between IE and recent dental procedures [4]. However, the analysis assessed the risks, including studies with intermediate and non-invasive dental procedures. This ambiguity poses a challenge, given that existing clinical guidelines predominantly address the risk associated with invasive dental procedures, leaving a gap in guidance for the full spectrum of dental care. In addition, studies on the global health landscape have documented a rising incidence of IE over the last three decades across numerous regions [5–7]. Eventually, the growing prevalence of IE poses significant implications for healthcare systems globally, leading to increased financial costs and resource allocations due to the necessity for surgical management and extended hospital stays in cases of severe IE. [8].

At present, the effectiveness of antibiotic prophylaxis (AP) for preventing IE is not sufficiently supported by scientific evidence due to the absence of randomized controlled trials (RCTs). The conduct of such studies is complicated by ethical considerations and the rarity of the disease. This emphasizes the importance of investigating the risk of IE development following invasive dental procedures, which may clarify the causal relationship and either support or question the necessity for AP prior to these interventions.

Therefore, the primary objective of this systematic review was to consolidate and analyze the existing evidence regarding the risk of IE associated with dental procedures, including both invasive and non-invasive procedures, incorporating a detailed subgroup analysis by procedure type. The meta-analysis specifically aimed to determine if certain dental procedures, such as tooth extraction and oral surgery, significantly increase the risk of IE compared to others.

## Methods

### Search Strategy and Selection Criteria

This systematic review and meta-analysis were performed according to the PRISMA guidelines. The search was conducted across PubMed, Google Scholar and Cochrane databases, imposing no restrictions on publication date or language. Our search strategy employed a combination of keywords and MeSH terms, focusing on “infective endocarditis,” “invasive dental procedures,” “dental procedures,” “tooth extraction,” “scaling,” “endodontic treatment,” “oral surgery” and “periodontal treatment.” These terms were combined using Boolean operators to refine the search parameters effectively. The detailed search strategy is available in the supplementary (Supplementary Material). The most recent search was conducted on 13 December 2023.

For this research, we carefully defined our inclusion criteria using the PICOS framework to ensure a structured and comprehensive approach.

Population: We focused on individuals aged 15 years and older, aligning with the World Health Organization’s age categorization for adolescents and adults.

Intervention: The study considered invasive dental procedures as the primary intervention, specifically including tooth extraction, scaling, endodontic treatment, oral surgery, and periodontal treatment.

Comparison: Our comparison group comprised individuals without IE and utilized control periods within case-crossover studies to facilitate accurate assessments.

Outcomes: We examined the incidence of IE subsequent to invasive dental procedures, insisting on a minimum follow-up duration of 3 months to ensure consistency and comparability across studies.

Study Design: Our review included both prospective and retrospective observational studies, encompassing case-control, cross-sectional, cohort, and case-crossover designs to capture a broad spectrum of evidence.

Exclusion criteria were applied to studies focusing on non-dental invasive procedures, as well as to case series, case reports, and animal studies. Two investigators (ZhK and AA) independently evaluated the titles and abstracts and screened the full text of the articles for final inclusion. Any disagreement in the search and selection processes was resolved by consensus.

### Data Analysis

A customized data extraction form was used to extract the study characteristics, including first author and publication year, PMID, design, country, data sources, study period, cases and controls, population, sample size, identification of IE, cardiac condition of patients, intervention (type of dental procedures), comparison and outcomes. Data extraction was performed by two reviewers (ZhK and AA) and verified by two others (MK and TB). The quality assessment was carried out by two independent reviewers (ZhK and MK).

The cohort and case-control studies were assessed based on the Newcastle-Ottawa Scale (NOS) checklist to Agency for Healthcare Research and Quality (AHRQ) standards (good–3 or 4 stars, fair–2 stars, and poor–0 or 1 star). The star scoring system for prospective and cross-sectional studies is a maximum of nine points. Selecting the appropriate assessment tool for case-crossover design was difficult as the study was hybrid of case-control study and crossover design [9].The ROBINS-I tool was used as a more appropriate assessment tool for case-crossover designs [10].

To facilitate a comprehensive analysis, we systematically organized the included studies into categories based on the level of procedural invasiveness as detailed in their methodologies:

Invasive Dental Procedures: The first category encompasses studies that exclusively focus on invasive dental procedures. These are defined in accordance with the American Heart Association (AHA) guidelines, which include interventions involving manipulation of the gum tissue and oral mucosa [11]. Examples of such procedures include tooth extraction, scaling, endodontic treatment, oral surgery, and periodontal treatment. This grouping allowed us to isolate the impact of procedures recognized for their potential to introduce bacteria into the bloodstream, warranting AP.

Dental Procedures: The second category broadens the scope to include studies that investigate not only invasive dental procedures but also intermediate and non-invasive dental procedures. These latter procedures typically do not require AP and encompass a wide range of dental care activities, such as restorative dental work, fluoride treatments, routine dental exams, and radiographic evaluations (Table 2).

To explore deeper into the impact of invasive dental procedures on the incidence of IE, we performed a detailed subgroup analysis, stratifying by the IE-risk profile of the patient populations as delineated by the American Heart Association (AHA) [11] and the European Society of Cardiology (ESC) [12] guidelines:

High IE-risk Individuals: This subgroup includes studies focusing on patients characterized by a heightened risk of IE. Such individuals typically have prosthetic heart valves, a history of IE, or congenital cyanotic heart disease. This categorization allowed us to specifically assess the risk associated with invasive dental procedures in a population already at elevated risk for IE.

General Cardiac Individuals: For studies that did not distinguish patients based on their specific IE risk, we created a broader category termed “General cardiac individuals.” This grouping captures the general cardiac patient population without specifying their IE risk level (Table 2).

In addition to stratifying by patient risk groups, we conducted a further subgroup analysis focusing on the type of invasive dental procedure performed, including “tooth extraction,” “scaling,” “endodontic treatment,” “oral surgery,” and “periodontal treatment.” This approach allowed for a detailed examination of the potential differential risk these various dental interventions may pose for the development of IE.

The primary outcome was the incidence of IE following only invasive dental procedures. This analysis was specifically segmented to consider not only the overall incidence following any invasive dental procedure but also to differentiate among the varied types of procedures, such as tooth extraction, scaling, endodontic treatment, oral surgery, and periodontal treatment. As a secondary aim, the study also explored the incidence of IE following a broader range of dental procedures, encompassing both invasive and non-invasive interventions.

For dichotomous outcomes, we used the Mantel-Haenszel fixed-effect method. For the generic inverse variance data type, we used the inverse-variance method and independently calculated the standard error (SE) from 95% confidence interval (CI) and log (OR). The heterogeneity of the studies was assessed by the I^2^ test. According to the Cochrane Handbook interpretation of results was heterogeneity not be important if 0%–40%, moderate 30%–60%, substantial 50%–90%, considerable 75%–100%. With substantial heterogeneity, we used a random-effects method. A sensitivity analysis was performed by repeating the primary analysis of removing one of the studies when significant heterogeneity was present.

The forest plot was created in Review Manager (RevMan) software Version 5.4. (Nordic Cochrane Centre, The Cochrane Collaboration, 2012, Copenhagen, Denmark).

This study is registered with PROSPERO, number CRD42023488546.

## Results

**FIGURE 1:**
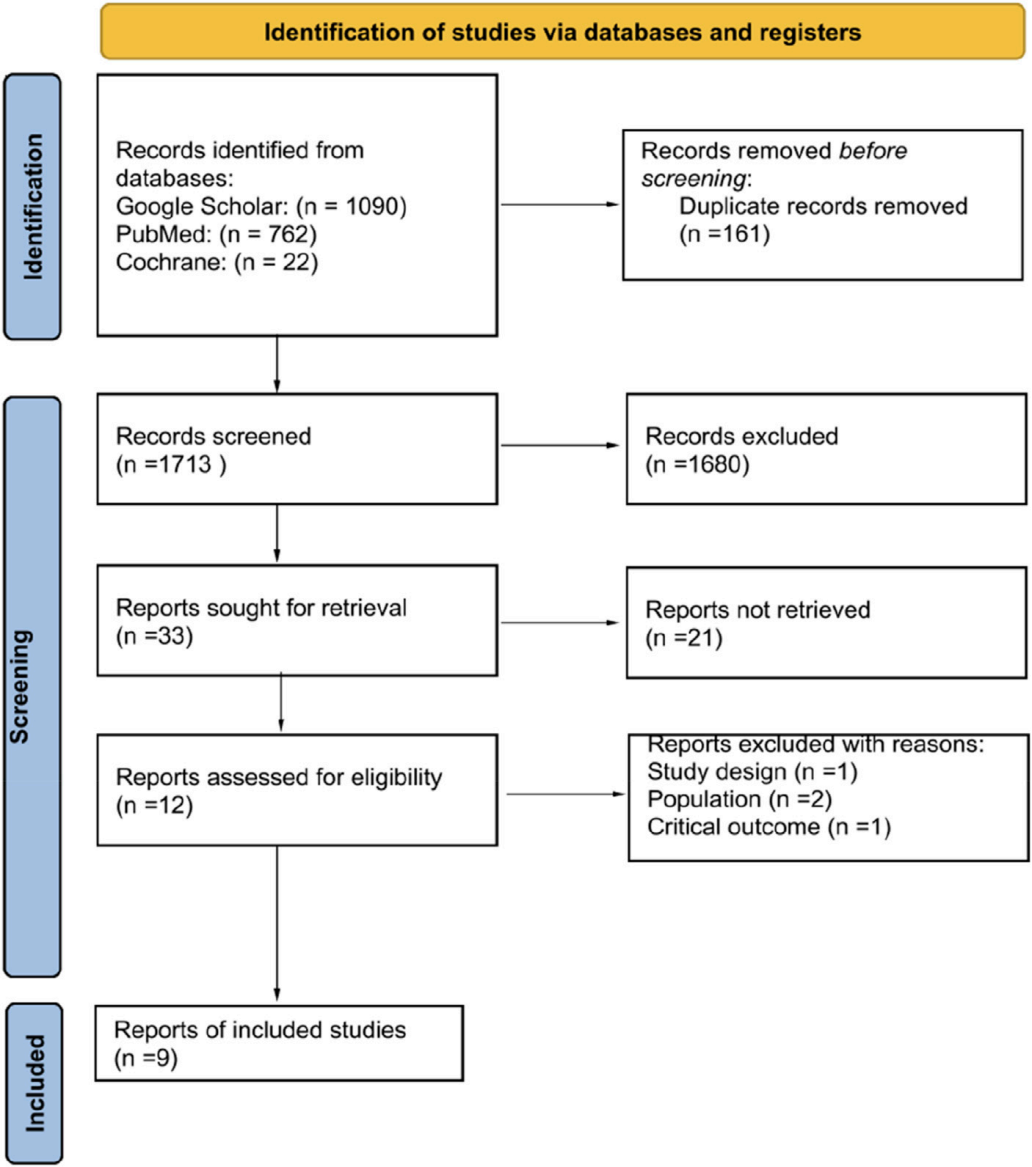
Study selection process (Kazakhstan. 2023).

The selection process for studies included in our systematic review and meta-analysis is depicted in the PRISMA flow diagram (Figure 1). Initially, our search across three databases yielded a total of 1,874 records. Following the removal of duplicates, 1,713 records underwent screening based on their titles and abstracts. This preliminary assessment led to the identification of 33 articles as potentially eligible, which were then subjected to a full-text review. Finally, nine articles were included into our systematic review and meta-analysis [13–21].

An overview of the characteristics of the included studies are provided in Tables 1, 2. They consisted of seven case-crossover studies [13–19], three cohort studies [13–15], and two case-control studies [20, 21]. These studies were conducted in the US (three studies), France (two studies), Taiwan (two studies), the UK and Israel. The sample size varied from 170 to 14 731 patients, and publication years ranged from 1995 to 2023. A quality assessment of the studies revealed that six achieved a rating of >5 stars out of a maximum of 9, indicating a generally high quality, while the case-crossover studies were classified as having a moderate risk of bias, as detailed in the Supplementary (Supplementary Table S1; Supplementary Figures S1–S2).

**TABLE 1:** The main characteristics of the studies (Kazakhstan. 2023).

	Author, year	Country	Design	Data source	Study time period	Population age	Total number of patients	Cases	Controls	Dental procedures associated with the risk of IE?
1	Lacassin, 1995	France	Case-control	Hospitals (medical facilities)	1990–1991	>15 years	171	IE	without IE	No, (scaling and root canal treatment showed a trend risk)
2	Strom, 1998	US	Case-control	Hospitals	1988–1990	>18 years	273	IE	without IE	No
3	Porat, 2008	Israel	Case-crossover	Medical Centers	2003–2005	NA	170	the 3-month preceding IE	during earlier other 3-month periods	No
4	Chen, 2015	Taiwan	Case-crossover	The Health Insurance Database	1999–2012	>18 years	713	the 3-month preceding IE	during earlier other 3-month control periods	No
5	Chen, 2018	Taiwan	Case-crossover	The Health Insurance Database	2005–2011	<20 years of age or >100 years	9 210	the 3 months preceding IE	during earlier other 3-month control periods	No
6	Tubiana, 2017	France	Case-crossover and Cohort	The French National Health Insurance	2008–2014	>18 years	138 876 for cohort and 846 for case control	the 3 months preceding IE for cohort IDP	during earlier other 3-month after washout periods; for cohort non-IDP	No
7	Thornhill, 2022	UK	Case-crossover and Cohort	The Hospital Episode Statistics database	2010–2016	>18 years	14 731	the 3-month preceding IE	during earlier other 3-month after washout periods	Yes, a significant IE association following extractions/surgical tooth removal
8	Thornhill, 2022	US	Case-crossover and Cohort	Commercial /Medicare Supplemental database	2000–2015	≥18 years	3,774	the 4-month preceding IE for cohort IDP	12-month control period (months 5–16); for cohort non-IDP	Yes, a significant IE association following tooth extractions and oral surgery
9	Thornhill, 2023	US	Case-crossover and Cohort	Commercial/Medicare Supplemental database	2000–2015	≥18 years	2,647	the 4 months preceding IE for cohort IDP	12-month control period (months 5–16); for cohort non-IDP	Yes, a significant IE association following tooth extractions and oral surgery

IE, infective endocarditis; IDP, invasive procedures; Non-IDP, Non-invasive procedures; NA, not available.

**TABLE 2:** The main characteristics of patients and type of dental procedures (Kazakhstan. 2023).

	Author, year	PMID	Identification of IE	Cardiac condition of patients	Risk groups	Type of dental procedures	The invasiveness of dental procedures
1	Lacassin, 1995	8682034	Von Reyn’s criteria	Native valve disease, prosthetic valve disease and no know cardiac disease	General cardiac individuals	Extraction, scaling, root canal treatment and any dental procedure	DP
2	Strom, 1998	9841581	NA	Mitral valve prolapse, congenital heart disease, history of rheumatic fever with heart involvement, prosthetic heart valve, previous episode of endocarditis, or other valvular heart disease.	General cardiac individuals	Dental hygiene care, filling, periodontal treatment, restorative dentistry, extraction, endodontic treatment, treatment of tooth abscess, mouth gingival surgery, fluoride treatment, other dental procedures, any invasive dental procedures, any dental procedures	DP
3	Porat, 2008	8797944	Duke criteria	Underlying cardiac conditions, native valve defects, prosthetic valves, cardiac pacemakers	General cardiac individuals	Scaling, root planing, endodontic treatment, dental extraction, implant placement, initial placement of orthodontic bands, and other surgical and nonsurgical procedures	DP
4	Chen, 2015	26512586	ICD-9-CM	Valvular heart disease, ischemic heart disease	General cardiac individuals	Tooth extraction, surgery, dental scaling, periodontal treatment, and endodontic treatment	IDP
5	Chen, 2018	29674326	ICD-9-CM	Rheumatic heart disease or valve replacement	General cardiac individuals	Dental scaling and root planing, simple extraction, complicated extraction, odontectomy in both simple case and complicated case, and periodontal surgery	IDP
6	Tubiana, 2017	28882817	ICD-10	Replacement of prosthetic heart valves	High IE-risk individuals	Consistent with 2015 European guidelines IDP (invasive when they required manipulation of the gingival or periapical region of the teeth or perforation of the oral mucosa (excluding local anaesthetic injection)) and non-IDP (other dental procedures)	DP (IDP and Non-IDP)
7	Thornhill, 2022 (UK)	36137742	ICD-10	Individuals at high IE risk, moderate risk and at low/unknown risk	General cardiac individuals	Extractions/surgical tooth removal, scaling and gingival procedures, other oral surgical procedures	IDP
8	Thornhill, 2022 (US)	35987887	ICD-9	Individuals at high IE risk, moderate risk and at low/unknown risk	High IE-risk individuals	IDP that involve manipulation of gingival tissue or the periapical region of the teeth, or perforation of the oral mucosa, endodontic procedures	IDP
9	Thornhill, 2023 (US)	37103475	ICD-9	Individuals at high IE risk, moderate risk and at low/unknown risk	High IE-risk individuals	IDP that involve manipulation of gingival tissue or the periapical region of the teeth, or perforation of the oral mucosa, endodontic procedures	IDP

IE, infective endocarditis; IDP, invasive procedures; Non-IDP, Non-invasive procedures; DP, Dental procedures (including invasive dental procedures, intermediate dental procedures and non-invasive dental procedures); ICD, International Classification of Diseases; NA, not available.

**FIGURE 2:**
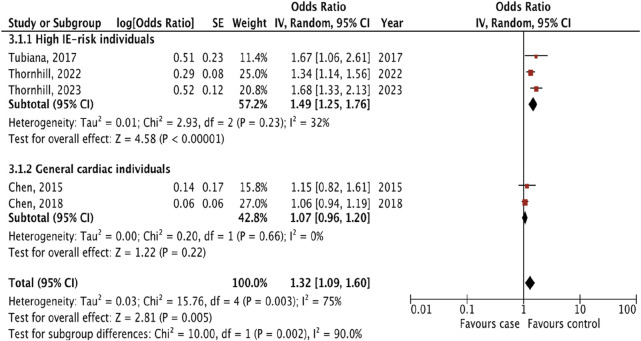
The pooled effect of the risk of infective endocarditis following “invasive dental procedures” (Kazakhstan. 2023).

Five studies that examined the risk of IE following only invasive dental procedures were included in the analysis [13–15, 17, 18]. The overall effect showed the presence of the risk of IE incidence following invasive dental procedures (OR 1.32, 95% CI 1.09 to 1.60; p = 0.005; I^2^ = 75%, Figure 2).

To more accurately assess the risk of IE, we performed a subgroup analysis by dividing the studies in two groups: 1) high IE-risk individuals, and 2) general cardiac individuals. Three studies [13–15] included patients in high IE-risk groups, but Tubiana et al [13] studied patients only with prosthetic heart valve disease. Two studies [17, 18] did not divide patients into risk groups. The result of subgroup analyses showed that the risk of IE following invasive dental procedures was presented only for patients in the high IE-risk group (OR 1.49, 1.25 to 1.76; p < 0.00001; I^2^ = 32%), and there were significant differences between subgroups (p = 0.002; I^2^ = 90%).

**FIGURE 3:**
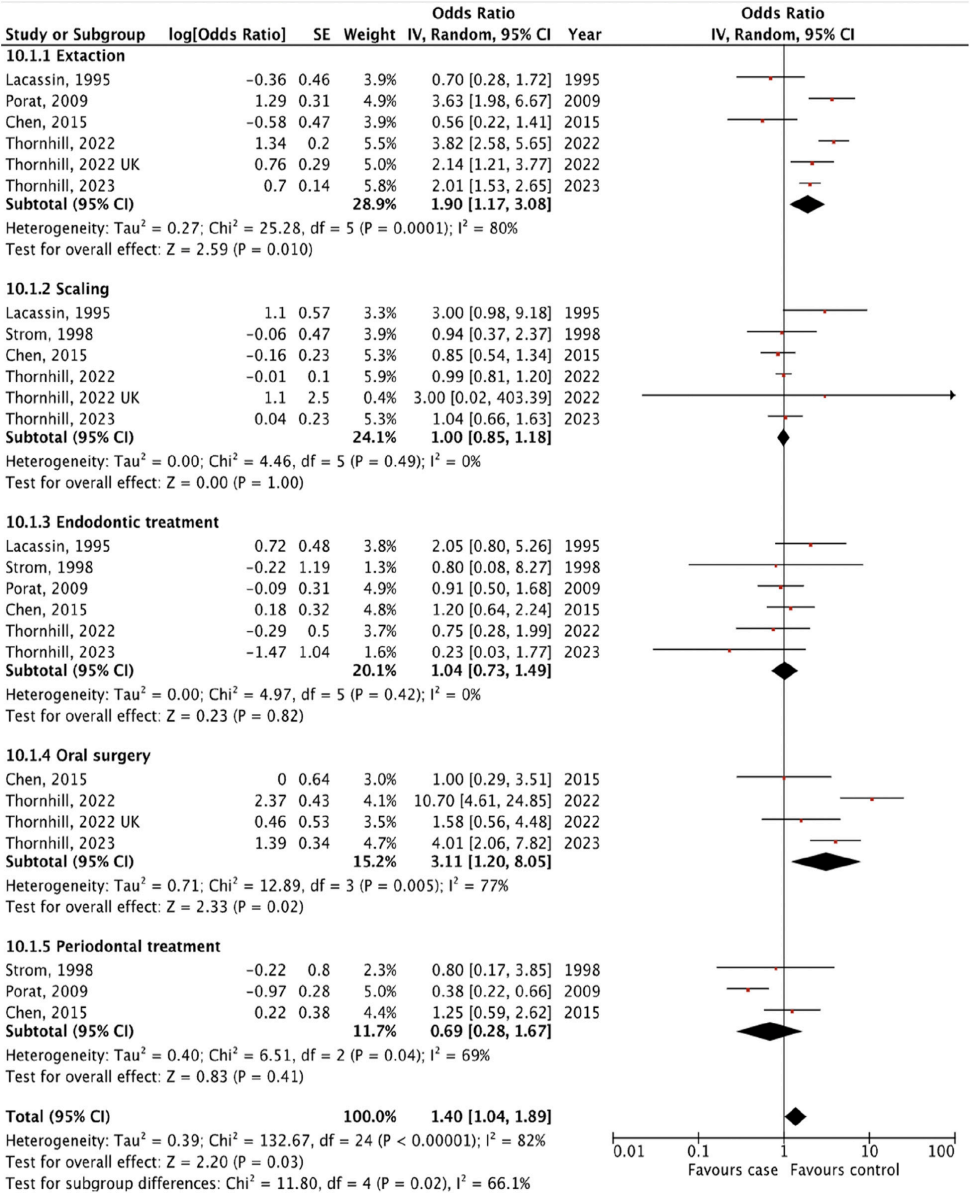
The pooled effect of subgroup analysis of the risk of infective endocarditis following the different types of “invasive dental procedures” (Kazakhstan. 2023).

We identified seven studies [14–16, 18–21] that provided an estimated risk of IE following different types of invasive dental procedures including tooth extraction, scaling, endodontic treatment, oral surgery and periodontal treatment (Figure 3).

We revealed the strongest association between IE and tooth extraction (OR 1.90, 95% CI 1.17 to 3.08; p = 0.010; I^2^ = 80%; Figure 3) and oral surgery (OR 3.11, 95% CI 1.20 to 8.05; p = 0.02; I^2^ = 77%; Figure 3). On the other hand, there was no significant association between IE and invasive dental procedures such as tooth scaling (OR 1.00, 95% CI 0.85 to 1.18; p = 1.00; I^2^ = 0%; Figure 3), endodontic treatment (OR 1.04, 95% CI 0.73 to 1.49; p = 0.82; I^2^ = 0%; Figure 3), and periodontal treatment (OR 0.69, 95% CI 0.28 to 1.67; p = 0.41; I^2^ = 69%; Figure 3).

The analysis of the collective impact of invasive dental procedures across individual studies revealed a statistically significant pooled effect, with an OR of 1.40 (95% CI 1.04 to 1.89; p = 0.03; I^2^ = 82%), indicating a notable risk increase (Figure 3). This analysis also highlighted significant variability among the studies, as evidenced by a p-value of 0.02 and an I^2^ of 66% for subgroup differences. To enhance the reliability of our findings, one with the lowest quality ratings was excluded in a sensitivity analysis. According to the results of the research quality assessment, the study [21] was awarded 5 stars out of a maximum possible 9 stars. This adjustment did not significantly alter the results, with the revised pooled effect OR for the remaining five studies standing at 1.46 (95% CI 1.06 to 2.00, p = 0.02; I^2^ = 84%) (Supplementary Table S2).

**FIGURE 4:**
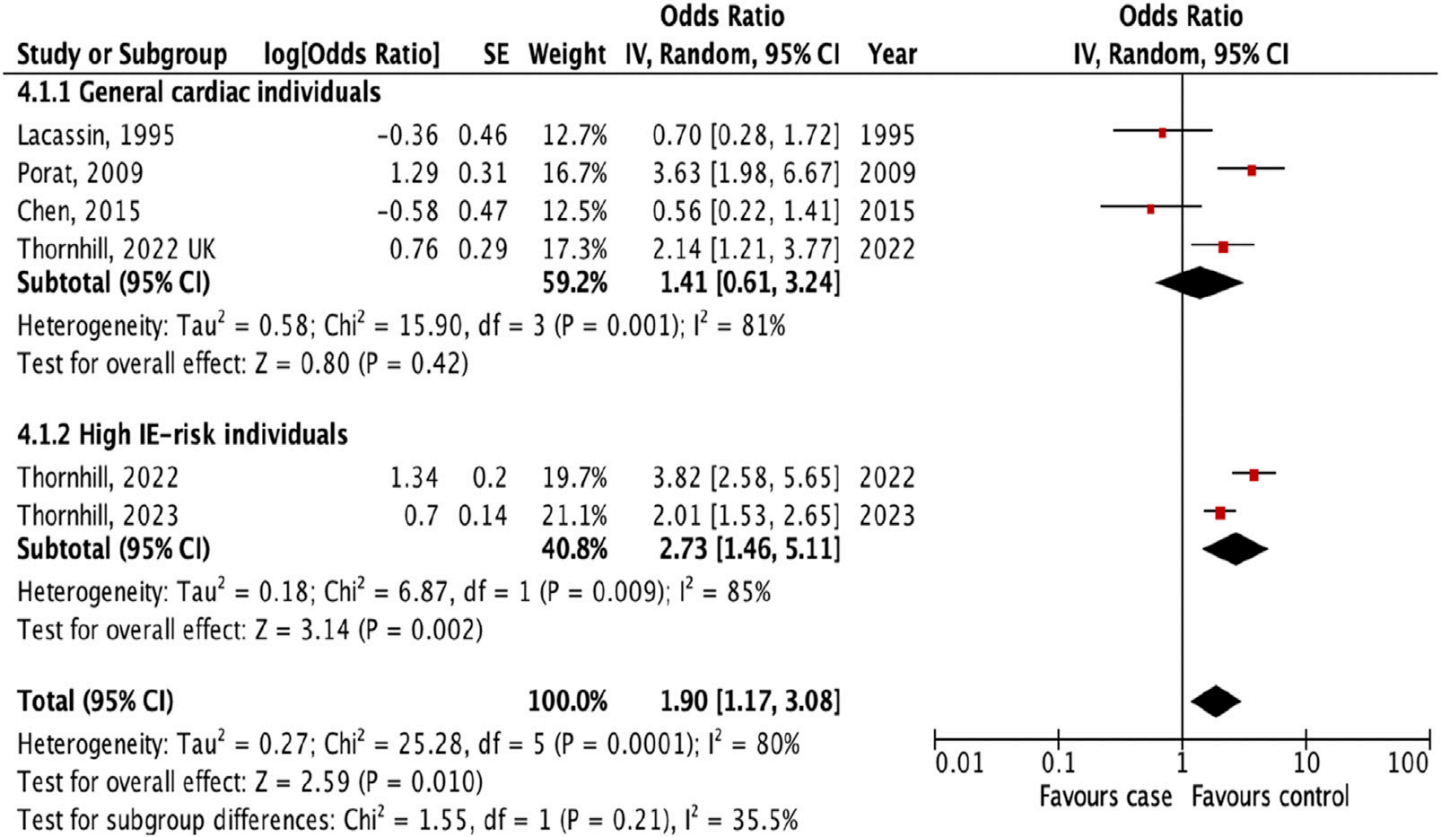
The pooled effect of subgroup analysis of the risk of infective endocarditis following tooth extraction (Kazakhstan. 2023). All corresponding plots are provided in the Supplementary Figures S4–S6.

**FIGURE 5:**
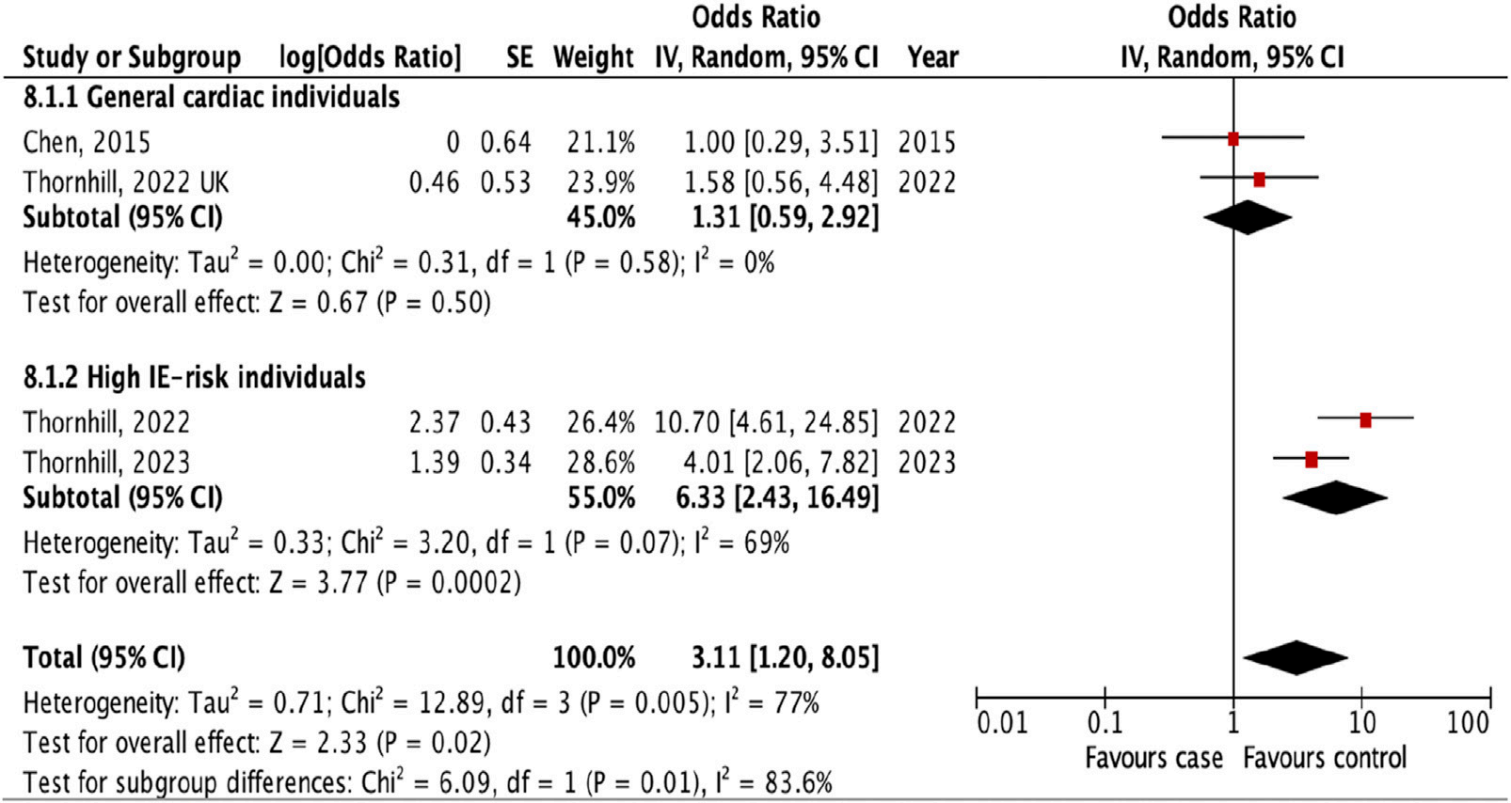
The pooled effect of subgroup analysis of the risk of infective endocarditis following oral surgery (Kazakhstan. 2023). All corresponding plots are provided in the Supplementary Figures S4–S6.

Additional analyses were done to investigate the prevalence of IE following different types of invasive dental procedures among high IE-risk individuals and general cardiac individuals. The result showed that the risk of IE was higher after tooth extraction (OR 2.73, 95% CI 1.46 to 5.11; p = 0.002; I^2^ = 85%; Figure 4) and oral surgery (OR 6.33, 95% CI 2.43 to 16.49; p = 0.0002; I^2^ = 69%, Figure 5) only for high IE-risk individuals.

**FIGURE 6:**
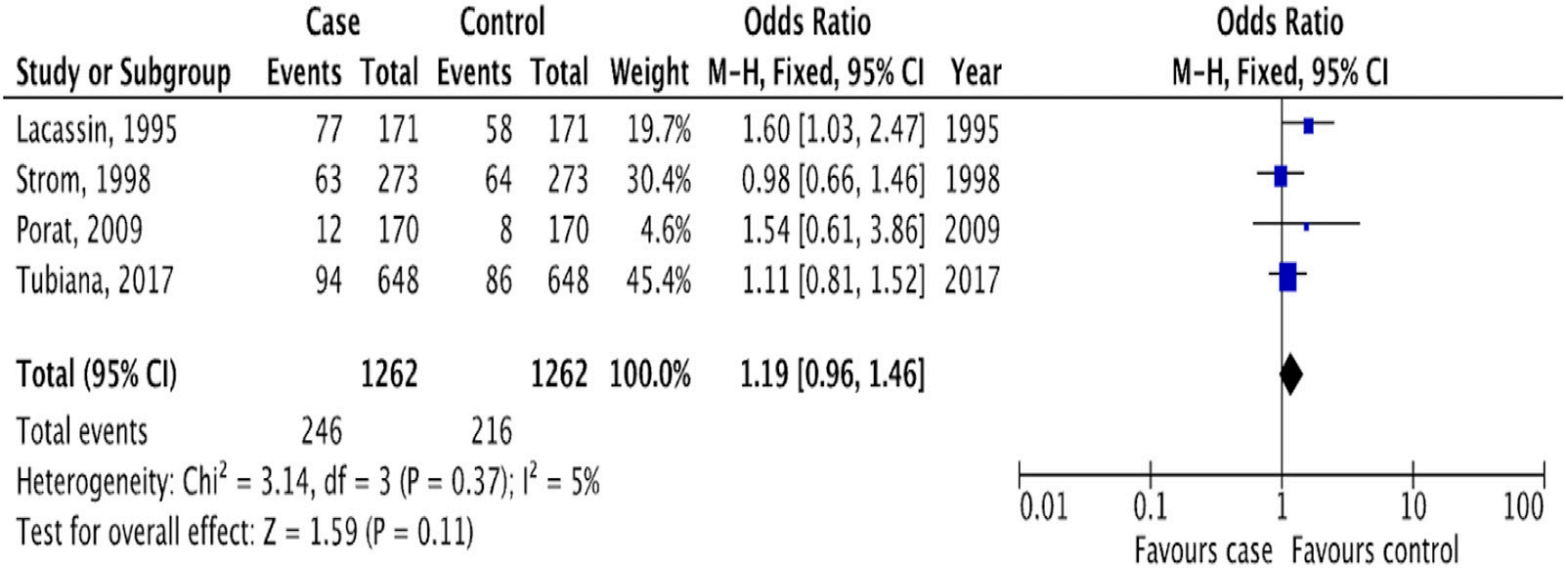
The pooled effect of the risk of infective endocarditis following “dental procedures” (Kazakhstan. 2023). All corresponding plots are provided in the Supplementary Figure S3.

In evaluating the risk of IE associated with dental procedures, our analysis included studies that reported outcomes for all types of dental interventions, without distinguishing by invasiveness level. This included four studies [13, 19–21] that ranged in procedures from fluoride treatments to oral surgery, collectively categorized as “dental procedures.” The risk assessment was based on the incidence of IE among patients who had undergone any dental procedure within 3 months prior to the onset of IE. Our findings indicated no significant association between dental procedures and the risk of IE, with an OR of 1.19 (95% CI 0.96 to 1.46; p = 0.11; I^2^ = 5%, Figure 6). Further analysis confirmed the stability of this outcome (OR 1.19, 95% CI 0.96 to 1.49; p = 0.12; I^2^ = 11%, Supplementary Figure S3), underscoring the lack of a significant link between undergoing dental procedures and the subsequent risk of developing IE.

## Discussion

To our knowledge, this systematic review and meta-analysis represents the first comprehensive effort to investigate the relationship between IE and invasive dental procedures, as well as to dissect the associations pertaining to each specific type of invasive dental intervention. The association between IE and invasive dental procedures was observed exclusively among patients identified as belonging to high IE-risk categories. This underscores the particular vulnerability of this group to IE post-invasive dental care.

Among the various invasive dental procedures examined, tooth extraction and oral surgery emerged as having the most significant association with IE incidence, again specifically within the high IE-risk patient cohort. This finding highlights the need for increased awareness and potentially tailored prophylactic measures for these procedures in such high-risk individuals. When considering the wider category of “dental procedures,” which includes invasive, intermediate, and non-invasive dental interventions, no significant correlation with the prevalence of IE was detected. This indicates that the risk of IE is not uniformly elevated across all types of dental care but is instead confined to specific invasive procedures among patients already at increased risk.

The association between IE and “invasive dental procedures” has not been previously investigated. The individual observational studies [13, 17–21] did not reveal the risk of IE following invasive dental procedures compared with controls, except in Thornhill’s studies [14–16] (Table 2). In our study, the pooled effect showed that the risk was significantly higher in patients who underwent invasive dental procedures 3 months before disease onset (OR 1.32, 95% CI 1.09 to 1.60; p = 0.005; I^2^ = 75%; Figure 2). However, the results must be interpreted with caution due to the high heterogeneity due to sample size variability from 648 to 9120 [13, 18]. The investigation of the subgroups allowed us to study the difference between high IE-risk individuals and general cardiac individuals, regardless of the patient’s risk. According to the results, the statistical significance was in the high IE-risk individuals (OR 1.49, 1.25 to 1.76; p < 0.00001) compared with general cardiac individuals (OR 1.07, 0.96 to 1.20; p = 0.22). Our results strongly support the 2021 AHA and the 2023 ESC guidelines, which partially restricted antibiotic prescribing to a high-risk group only before invasive dental procedures, including dental extractions, oral surgery procedures, and procedures requiring manipulation of the gingival or periapical region of the teeth [11, 12]. But they are not supportive of the UK National Institute for Health and Care Excellence (NICE) guidance recommending against the use of AP.

It is important to note that while our study found a link between invasive dental procedures and IE, this does not prove that AP is effective. More research is needed to evaluate its effectiveness, but our findings suggest that AP might help reduce the risk of developing IE.

The subgroup evaluation allowed us to conduct a more detailed analysis of the association between IE and each type of invasive dental procedure. We found a strong association between IE and tooth extraction (OR 1.90, 95% CI 1.17 to 3.08; p = 0.010; Figure 3) and oral surgery (OR 3.11, 95% CI 1.20 to 8.05; p = 0.02; I^2^ = 77%; Figure 3). We also examined the association between IE and tooth extraction and oral surgery between high IE-risk patients and general cardiac patients. According to the results, patients were at risk of developing IE after tooth extraction and oral surgery procedures only in high IE-risk individuals (OR 2.73, 95% CI 1.46 to 5.11; p = 0.002; I^2^ = 85%; Figure 4) and (OR 6.33, 95% CI 2.43 to 16.49; p = 0.0002; I^2^ = 69%, Figure 5), respectively.

Our results are consistent with the study by Thornhill et al [14–16] which identified an increased incidence of invasive dental procedures associated with a high risk for IE, especially following tooth extraction and oral surgery. This association can be explained by the fact that the alveolus following tooth extraction and oral surgical interventions is more susceptible to bacteremia. Our results are also confirmed by a recent study [4, 22], which found that the current high prevalence of the causative microorganism is *Streptococcus*, especially S. viridans which are dominant members of the resident oral microbiota and are a characteristic microorganism only for odontogenic infections.

Our analysis did not identify a significant association between IE and “dental procedures” including all types of dental procedures, not only invasive dental procedures. This contrasts with a recent study by Lean et al [4] which reported a significant association between IE and recent dental procedures. The discrepancy between these results can be attributed to differences in research methodologies. Unlike the previous review, our study differentiated between types of dental procedures based on their invasiveness, incorporating studies that exclusively examined invasive dental procedures [13, 17, 18] and studies with all dental procedures combined [19–21] (Table 2). This distinction is crucial as AP recommendations currently apply only to invasive dental procedures, potentially introducing bias and complicating the interpretation of results.

Our study has several limitations. First, our systematic review and meta-analysis are limited to published evidence, absence RCTs on the effectiveness of AP. Second, we did not restrict the year of publication, leading to a wide range of study years (over the past 30 years) and significant variability in sample sizes (from 170 to 14 731 individuals) [14, 19], contributing to heterogeneity. Third, some studies reported missing data or excluded patients, such as Porat et al. [19], which analyzed data from only 98 of the 170 patients. Fourth, the predominance of case-crossover studies, which may overestimate results, potentially skews findings. Fifth, follow-up periods for assessing the incidence of IE varied across studies, although we aimed for uniformity by selecting a common period where possible. Sixth, the subgroup analysis for high IE-risk groups may be biased, particularly when including multiple studies from the same lead author or a single study with a disproportionately large sample size [14–16]. Seventh, from three cohort studies only Tubiana et al [13] met the criteria of the design. Eighth, it was impossible to assess publication bias for statistical methods of Egger’s test or visual inspection of funnel plots because there were fewer than 10 studies. Ninth, studies use different methods and codes to identify IE, for example, ICD-9 and ICD-10. Finally, A direct comparison between invasive and non-invasive dental procedures was not possible due to insufficient data.

The exact prevalence of IE worldwide is unknown as the data from epidemiological studies of IE are mainly collected from developed countries. Studies on the global burden of disease lack data from low- and middle-income countries, including the Central Asian region. Therefore, for a complete picture of the morbidity and mortality associated with IE, additional research is required in developing countries as well as in countries with economies in transition. It would be appropriate if future studies provided data on patients at risk with the evaluation of blood cultures and also provided the number of patients and the number of procedures separately for uniformity of results, as one patient may undergo multiple procedures. Given the limitations of this study, further well-designed studies are needed to evaluate the association between IE and invasive and non-invasive dental procedures.

## Conclusion

The evidence from our study indicates a clear association between the risk of IE and the performance of invasive dental procedures, particularly among patients classified within the high IE-risk group. Notably, among the range of invasive dental interventions, tooth extraction and oral surgery were identified as carrying the greatest risk for the development of IE in these high-risk individuals. This finding underscores the latest update of the 2023 ESC guidelines.

## Abbreviations

AHA, American Heart Association; AHRQ, Agency for Healthcare Research and Quality; AP, Antibiotic Prophylaxis; ESC, European Society of Cardiology; IE, Infective Endocarditis; NICE, National Institute for Health and Care Excellence.
