# Supplementary material for: Risk of Infective Endocarditis Following Invasive Dental Procedures: A Systematic Review and Meta-Analysis
Source: Public Health Rev. 2025 Jan 8;45:1607684. doi: 10.3389/phrs.2024.1607684 (PMC11750435; doi:10.3389/phrs.2024.1607684)

Supplemental-Methods:

Search strategy

PubMed

1. "Endocarditis"[Mesh]
2. "Endocarditis" [tw] OR "Bacterial endocarditis" [tw] OR "Infective endocarditis" [tw]
3. #1 OR #2
4. "Tooth Extraction"[Mesh] OR "Oral Surgical Procedures"[Mesh]
5. "dental procedures" [tw] OR "invasive dental procedures" [tw] OR"non invasive dental procedures" [tw]
6. "tooth extract*"[tw] OR "oral surg*"[tw] OR "endodontic treatment"[tw] OR "root canal treatment"[tw] OR "dental scaling"[tw] OR "periodontal treatment"[tw]
7. #4 OR #5 OR #6
8. #3 AND #7

Cochrane

#1 MeSH descriptor: [Endocarditis] explode all trees
#2 (Endocarditis):ti,ab,kw OR ("bacterial endocarditis"):ti,ab,kw OR ("infective endocarditis"):ti,ab,kw
#3 #1 or #2
#4 ("dental procedures"):ti,ab,kw OR ("invasive dental procedures"):ti,ab,kw
#5 #3 and #4

Table 1. Quality assessment for observational studies according to Newcastle-Ottawa Scale (NOS). (Kazakhstan. 2023).

Study	Selection				Comparability	Outcome			Total ★
	**Representativeness of the exposed cohort**	**Selection of the non-exposed cohort**	**Ascertainment of exposure**	**Demonstration that outcome of interest was not present at start of study**	**Comparability of cohorts based on the design or analysis controlled for confounders**	**Assessment of outcome**	**Was follow-up long enough for outcomes to occur**	**Adequacy of follow-up of cohorts**	
Tubiana, 2017			★		★★	★	★	★	6
Thornhill, 2022 US			★		★★	★	★	★	6
Thornhill, 2023 US			★		★★	★	★	★	6
**Study**	**Is the Case Definition Adequate?**	**Representativeness of the Cases**	**Selection of Controls**	**Definition of Controls**	**Comparability of Cases and Controls based on the Design or Analysis**	**Assessment of Exposure**	**The same method of ascertainment for cases and controls**	**Non-Response rate**	**Total ★**
Lacassin, 1995	★	★	★		★★	★		★	7
Strom, 1998	★	★	★			★		★	5

The Newcastle-Ottawa Scale (NOS) checklist to AHRQ standards (good–3 or 4 stars, fair–2 stars, and poor–0 or 1 star).

Table 2. Sensitivity analysis. (Kazakhstan. 2023).

	Primary analysis	Sensitivity analysis
Dental procedures	4 studies (n=1262)<br>(OR 1.19, 95% CI 0.96–1.46, p=0.11)<br>I^2=5%	3 studies (n=989)<br>(OR 1.27, 95% CI 1.00–1.63, p=0.05)<br>I^2=0%
Invasive dental procedures: subgroup analysis	7 studies<br>(OR 1.40, 95% CI 1.04–1.89, p=0.03)<br>I^2=82%	6 studies<br>(OR 1.46, 95% CI 1.06–2.00, p=0.02)<br>I^2=84%
Scaling	6 studies<br>(OR 1.00, 95% CI 0.85–1.18, p=1.00)<br>I^2=0%	5 studies<br>(OR 1.00, 95% CI 0.85–1.18, p=0.98)<br>I^2=10%
Endodontic treatment	6 studies<br>(OR 1.04, 95% CI 0.73–1.49, p=0.82)<br>I^2=0%	5 studies<br>(OR 1.05, 95% CI 0.73–1.51, p=0.79)<br>I^2=19%
Periodontal treatment	3 studies<br>(OR 0.69, 95% CI 0.28–1.67, p=0.41)<br>I^2=69%	2 studies<br>(OR 0.67, 95% CI 0.21–2.14, p=0.50)<br>I^2=84%

Figure 1. ROBINS-I (risk of bias judgements in non-randomized studies of interventions) (Kazakhstan. 2023).

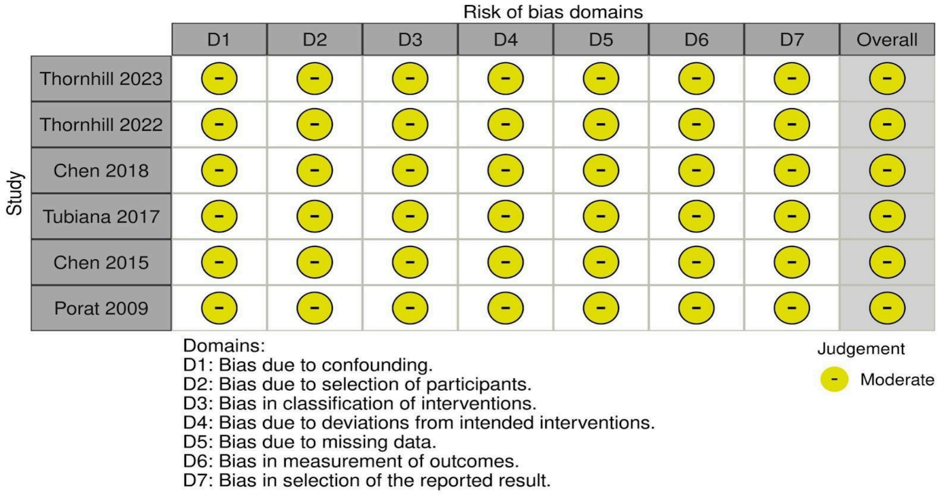

Figure 2. ROBINS-I (risk of bias judgements in non-randomized studies of interventions–summary plot) (Kazakhstan. 2023).

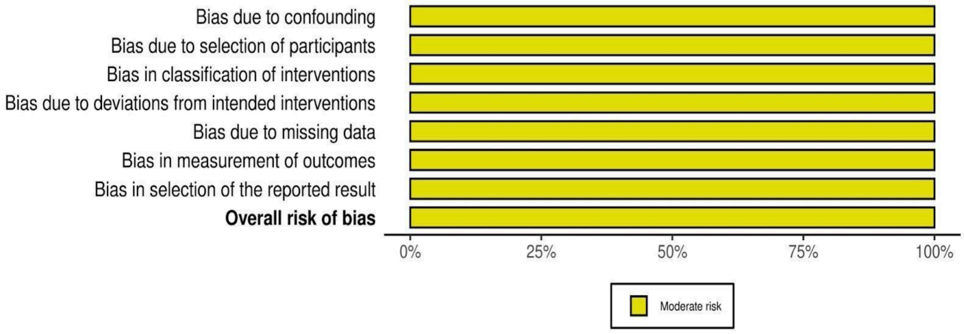

Figure 3. The pooled effect of the risk of infective endocarditis following “dental procedures” (Kazakhstan. 2023).

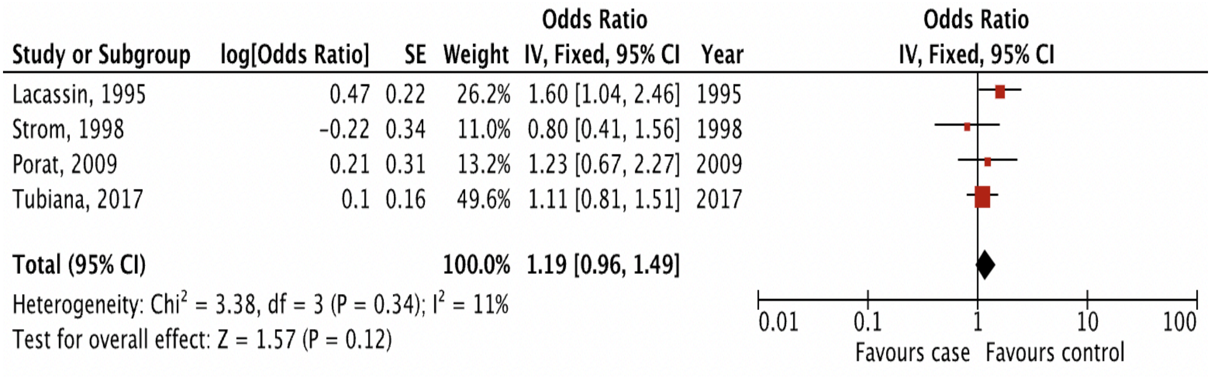

Figure 4. The pooled effect of the risk of infective endocarditis following endodontic treatment (Kazakhstan. 2023).

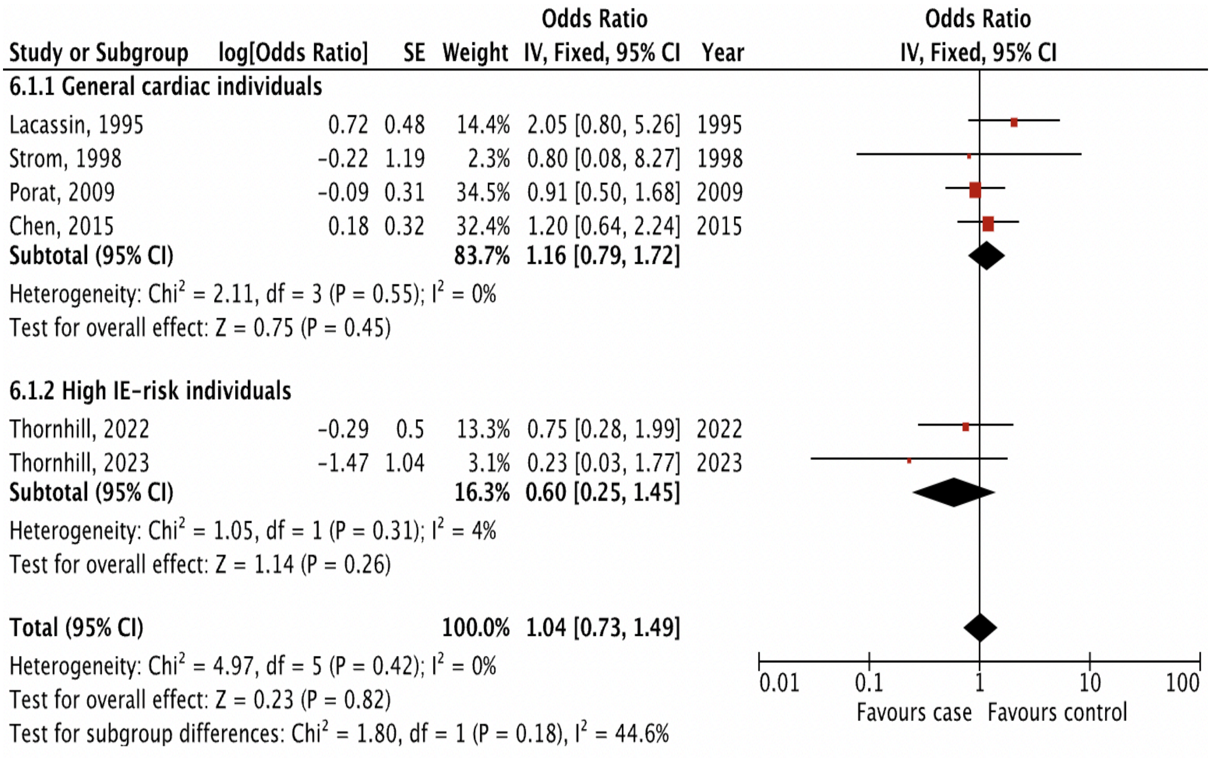

Figure 5. The pooled effect of the risk of infective endocarditis following periodontal treatment (Kazakhstan. 2023).

Study or Subgroup	log[Odds Ratio]	SE	Weight	Odds Ratio IV, Random, 95% CI	Year
Strom, 1998	-0.22	0.8	19.7%	0.80 [0.17, 3.85]	1998
Porat, 2009	-0.97	0.28	42.7%	0.38 [0.22, 0.66]	2009
Chen, 2015	0.22	0.38	37.6%	1.25 [0.59, 2.62]	2015
**Total (95% CI)**			**100.0%**	**0.69 [0.28, 1.67]**	
Heterogeneity: $Tau^2 = 0.40$; $Chi^2 = 6.51$, df = 2 (P = 0.04); $I^2 = 69\%$					
Test for overall effect: Z = 0.83 (P = 0.41)					

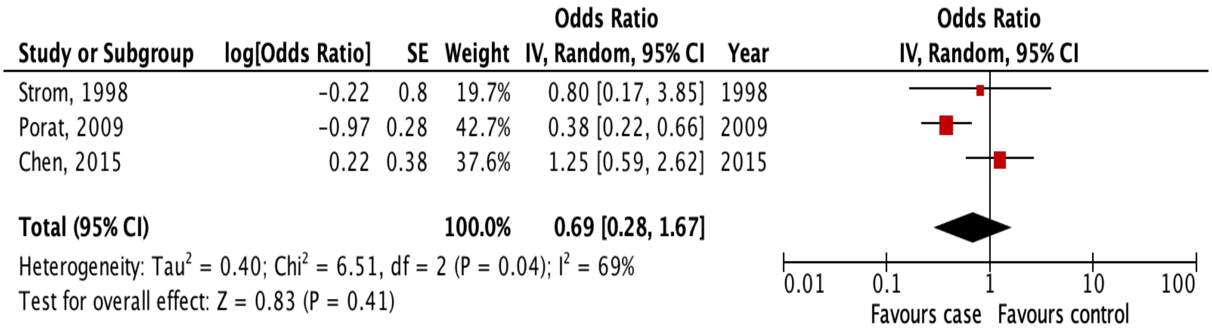

Figure 6. The pooled effect of the risk of infective endocarditis following scaling (Kazakhstan. 2023).

Study or Subgroup	log[Odds Ratio]	SE	Weight	Odds Ratio IV, Fixed, 95% CI	Year
**5.1.1 General cardiac individuals**					
Lacassin, 1995	1.1	0.57	2.1%	3.00 [0.98, 9.18]	1995
Strom, 1998	-0.06	0.47	3.1%	0.94 [0.37, 2.37]	1998
Chen, 2015	-0.16	0.23	13.0%	0.85 [0.54, 1.34]	2015
Thornhill, 2022 UK	1.1	2.5	0.1%	3.00 [0.02, 403.39]	2022
**Subtotal (95% CI)**			**18.3%**	**1.01 [0.69, 1.48]**	
Heterogeneity: $Chi^2 = 4.42$, df = 3 (P = 0.22); $I^2 = 32\%$					
Test for overall effect: Z = 0.05 (P = 0.96)					
**5.1.2 High IE-risk individuals**					
Thornhill, 2022	-0.01	0.1	68.7%	0.99 [0.81, 1.20]	2022
Thornhill, 2023	0.04	0.23	13.0%	1.04 [0.66, 1.63]	2023
**Subtotal (95% CI)**			**81.7%**	**1.00 [0.83, 1.19]**	
Heterogeneity: $Chi^2 = 0.04$, df = 1 (P = 0.84); $I^2 = 0\%$					
Test for overall effect: Z = 0.02 (P = 0.98)					
**Total (95% CI)**			**100.0%**	**1.00 [0.85, 1.18]**	
Heterogeneity: $Chi^2 = 4.46$, df = 5 (P = 0.49); $I^2 = 0\%$					
Test for overall effect: Z = 0.00 (P = 1.00)					
Test for subgroup differences: $Chi^2 = 0.00$, df = 1 (P = 0.96), $I^2 = 0\%$					

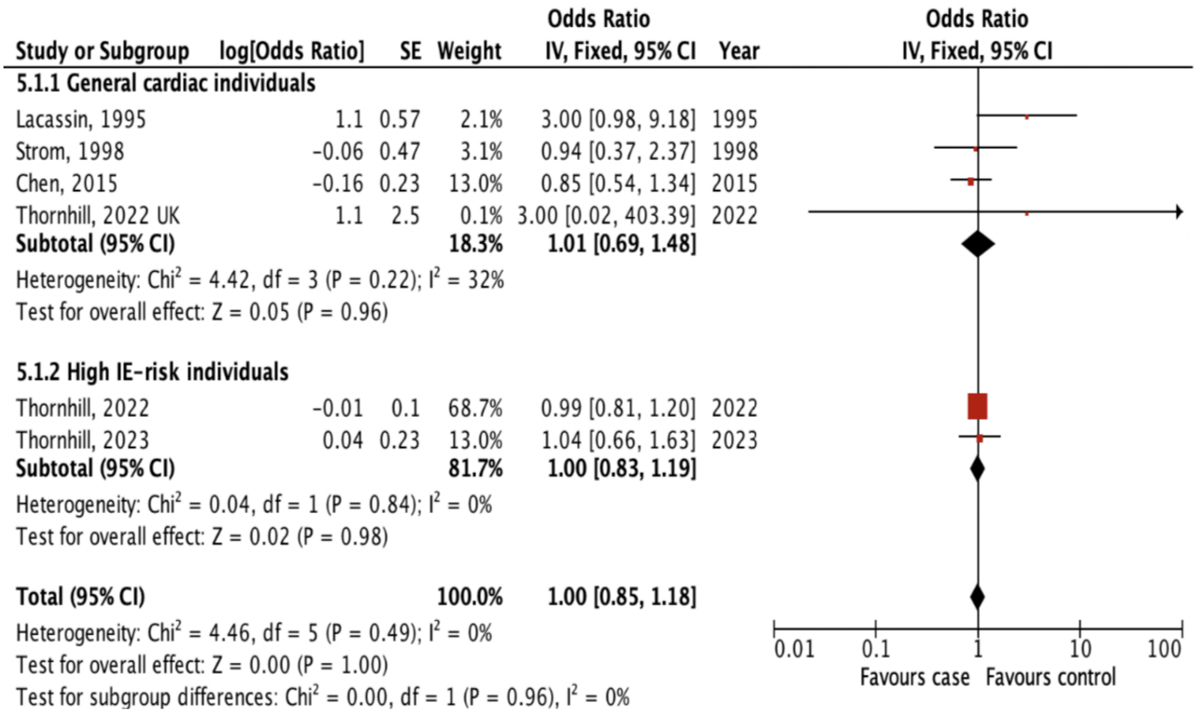

Supplement: Supplementary file 1 [file DataSheet1.pdf]
